# Ultrasound Detection of Patellar Fracture and Evaluation of the Knee Extensor Mechanism in the Emergency Department

**DOI:** 10.5811/westjem.2016.8.31051

**Published:** 2016-09-29

**Authors:** Kiersten Carter, Arica Nesper, Laleh Gharahbaghian, Phillips Perera

**Affiliations:** * Stanford University School of Medicine, Department of Emergency Medicine, Stanford, California; † Stanford/Kaiser- Emergency Medicine Residency Program, Department of Emergency Medicine, Santa Clara, California

## Abstract

Traumatic injuries to the knee are common in emergency medicine. Bedside ultrasound (US) has benefits in the rapid initial detection of injuries to the patella. In addition, US can also quickly detect injuries to the entire knee extensor mechanism, including the quadriceps tendon and inferior patellar ligament, which may be difficult to diagnose with plain radiographs. While magnetic resonance imaging remains the gold standard for diagnostic evaluation of the knee extensor mechanism, this can be difficult to obtain from the emergency department. Clinicians caring for patients with orthopedic injuries of the knee would benefit from incorporating bedside musculoskeletal US into their clinical skills set.

A 39-year-old male was brought into the emergency department (ED) after a washing machine fell onto his right knee. On physical examination, the patient had severe tenderness to palpation over the right knee. There was a significant amount of soft tissue swelling around the knee, a palpable joint effusion, and active right knee extension was markedly decreased. A bedside ultrasound (US) was performed using a 10-MHz linear transducer (FUJIFILM SonoSite Ultrasound) and showed severe cortical disruption of the patella, with associated hematoma on long-axis view (figure, video). The quadriceps tendon and patellar ligament, both critical structures for active knee extension, were found to be intact. A comminuted patellar fracture was confirmed on plain radiography, and intact quadriceps tendon and patellar ligament (also termed patellar tendon, but referred in this article as ligament) noted intra-operatively.

Previous literature describes the use of bedside musculoskeletal ultrasound (MSK US) to detect fractures. MSK US can be used for the diagnosis of long bone fractures, as well as to identify those fractures that may be difficult to identify on plain radiography. These include fractures of the sternum, ribs, scaphoid and metacarpals (and patella).^1^–^3^ MSK US is also very helpful in the evaluation for fracture in austere locations, or in practice environments with limited resources.^4^ Furthermore, MSK US can be particularly helpful in evaluating injuries to the entire extensor mechanism of the knee, especially when severe pain limits the physical examination.^5^,^6^ ^7^,^8^ US can potentially shorten the time to diagnosis and appropriate treatment of significant knee injuries, when compared with traditional methods.^9^

The high-frequency linear transducer is used for this MSK US application. A fracture is identified when there is a disruption in the normal continuous bright (hyperechoic) interface between the bone and soft tissue. Identification of a hypoechoic (dark) collection, suggestive of a hematoma, can also guide the clinician to the site of cortical disruption.^10^ In acute knee trauma, MSK US has been shown to have increased sensitivity, 94% versus 84%, over plain radiography in the diagnosis of fractures.^5^

To further assess the knee extensor mechanism using US, the probe is positioned in the long-axis configuration, cephalad to the superior pole of the patella and directly over the quadriceps tendon. It is then moved sequentially caudal over the patella down to the patellar ligament, allowing detailed assessment of these structures (see video).^11^

This case demonstrates the utility of bedside MSK US in the timely evaluation and management of patella fracture, and in assessment for associated knee ligamentous and tendon injuries. While magnetic resonance imaging has traditionally been the reference imaging standard for knee tendon and ligament injuries, US also has a high sensitivity for the diagnosis of these injuries.^12^ Therefore, MSK US is an increasingly important diagnostic modality for all healthcare providers who initially care for patients with acute knee injuries.

## Figures and Tables

**Figure:**
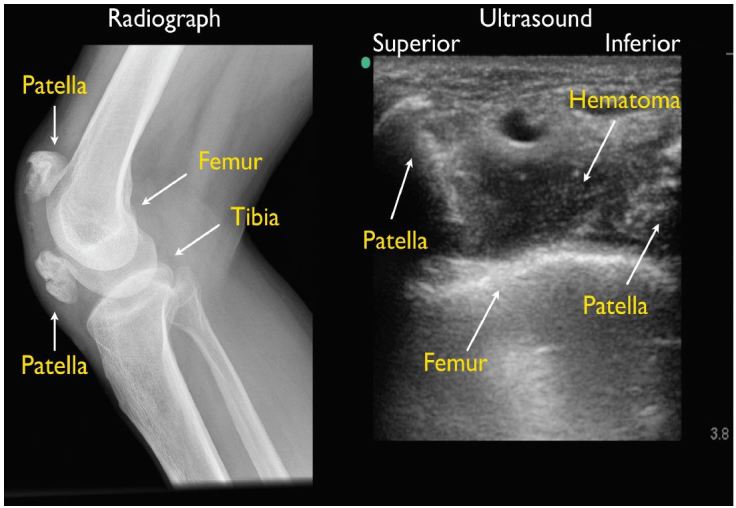
Patient’s knee radiograph and ultrasound.

**Video:** Ultrasound diagnosis of patellar fracture and evaluation of the extensor tendon mechanism of the knee. Insall-Salvati calculation of the relative distances of the patella and patellar ligament and the normal alignment of these structures is referenced in the video.^13^
